# Impact of obesity, structural severity and their combination on the efficacy of viscosupplementation in patients with knee osteoarthritis

**DOI:** 10.1186/s12891-019-2748-0

**Published:** 2019-08-17

**Authors:** Thierry Conrozier, Florent Eymard, Mickael Chouk, Xavier Chevalier

**Affiliations:** 1 Department of rheumatology, Hôpital Nord Franche-Comté, 100 route de Moval, CS 10490, Trevenans, 90015 Belfort, France; 2 Department of Rheumatology, Hôpital Henri Mondor, Creteil, France

**Keywords:** Knee, Osteoarthritis, Hyaluronic acid, Viscosupplementation, Obesity, X-rays, OARSI score, WOMAC

## Abstract

**Background:**

Obesity and radiological severity have been identified to be independent predictors of a low rate of response to viscosupplementation (VS), in patients with knee osteoarthritis (OA). Is that enough to formally refute VS in such patients in whom surgery is sometimes contraindicated?

**Objectives:**

To compare pain and function scores before and 6 months after knee VS, according to the weight status (obese versus non obese), the radiological severity (mild/moderate versus severe) and both combined.

**Methods:**

Post-hoc analysis of a prospective, double blind, randomized, multicentre trial, comparing 2 viscosupplements, in patients with symptomatic knee OA. Patients were classified according to body mass index (BMI < or ≥ 30 kg.^− 2^), OARSI radiological grade (1–2 versus 3) and OMERACT-OARSI response criteria (Yes/No). WOMAC between-group comparisons (obese versus non-obese, OARSI 1–2 versus 3, and both combined) in all patients and in OMERACT-OARSI Responders, were achieved using Mannn-Whitney U test.

**Results:**

One-hundred and sixty-six patients were analyzed: 28.3% were obese, 44% were OARSI grade 3, 42,2% were neither obese nor OARSI 3, whereas 14.5% were obese and OARSI 3. At baseline WOMAC pain score did not differ according to the patients sub-groups (*p* > 0.05). Six months after VS, WOMAC pain decreased significantly in all patient sub-groups (all *p* < 0.01). At month 6, WOMAC pain sub-score was significantly lower in non-obese than in obese patients (4.9 ± 4.1 versus 7.1 ± 4.9; *p* = 0.008) and in patients OARSI 1–2 versus 3 (4.8 ± 4.3 versus 6.4 ± 4.5; *p* = 0.009). However, in responder patients there was no difference in pain score and pain decrease related to the weight status and the radiological score.

**Conclusion:**

These results do not confirm our previous conclusions that recommended not performing VS in obese patients with severe knee OA. Although the chances of being a responder were much reduced in these patients, the benefit of patients who respond to treatment was similar to that of subjects with normal weight and mild/moderate OA. Different pain phenotypes, more than overweight and advanced disease, might be the main reason for the success or failure of VS.

## Background

Knee osteoarthritis (OA) is a major cause of pain and disability in subjects over 50 years with a significant impact on physical performance and quality of life. Standard conservative therapy for knee OA includes a combination of non-pharmacological and pharmacological approaches [1, 2]. However, none individually can be considered highly effective. In the early 90s, Balazs and Denlinger hypothesized that intra-articular (IA) injections of high molecular weight hyaluronic acid (HA) could restore the visco-elastic properties of the osteoarthritic synovial fluid (SF) that are altered in OA [3]. Twenty years later, viscosupplementation (VS) is widely used for treating patients with knee OA, not adequately improved by first line therapies [2]. Viscosupplementation is currently recommended by most scientific societies for the treatment of knee OA [2, 4–7]. The most recent meta-analyses and systematic reviews [8–14] concluded there was a clinically relevant efficacy of VS. One of them has ranked VS as the most effective treatment for knee OA [14]. Nevertheless, despite its wide use, the real efficacy of VS remains debatable [15, 16]. There are variable recommendations given across clinical guidelines [17]. For instance, the OARSI recommendations rated as “uncertain” the use of VS, based on contradictory conclusions among meta-analyses and conflicting results regarding safety [1]. The UK National Institute for Health and Care Excellence (NICE) and the American Association of Orthopaedi Surgeons (AAOS) recommended against the use of VS [18, 19]. To wind up the debate there is a huge need to identify the appropriate patients who may successfully respond to VS [20]. Only few trials have investigated the predictive factors of response to viscosupplementation [21–24]. Worst results have been reported in patients with advanced radiographic stages of the disease [21, 23]. Recently we demonstrated that radiological severity and obesity were two independent factors of VS failure [24]. We showed that the percentage of patients fulfilling the OMERACT-OARSI response criteria [25] was only 41.7% in patients with both marked joint space narrowing (JSN) and obesity, while it was 87.1% in those who did not have any of these two risk factors and 58.3% in subjects with only one. We concluded that VS should not be recommended in such patients who have few chances of successful treatment. Nevertheless, these results do not predict what may happen at an individual level. All clinicians performing VS have noticed that, in their daily clinical practice, some patients with very advanced stage of the disease and/or with morbid obesity have benefited in a sustainable way from the treatment. So, should we formally refute viscosupplementation in such patients in whom other therapeutic modalities (I.e. steroidal anti-inflammatory drugs -NSAIDs, corticosteroids or surgery) are often contra-indicated because of multiple co-morbidities?

The aim of the present work was to answer this question by assessing the impact of obesity, radiographic severity and their combination, not in term of response rate but in term of pain improvement and clinical status 6 months after IA-HA injections.

## Methods

The present study was a post-hoc analysis of a prospective, double-blind, randomized, multicenter and parallel-group trial, registered under the name HAV-2012 trial (N° EudraCT 2012-A00570–43). The primary goal of the study was to compare the efficacy and safety of 3 weekly injections of HANOX-M (HAppyVisc®, LABRHA SAS, Lyon, France) to BioHA (Euflexxa®, Ferring Pharmaceuticals, Parsippany, USA), according to a non-inferiority trial design, in patients with symptomatic knee OA [26, 27]. The study was performed in compliance with the principles of Good Clinical Practice (GCP) and the Declaration of Helsinki concerning medical research in humans and the country-specific regulations. Before enrolment, patients were asked to sign an informed consent form and were free to withdraw at any time for any reason. The patient informed consent form and the protocol, which complied with the requirements of the International Conference on Harmonisation (ICH), were reviewed and approved by the Ethics Committee of Lyon Sud-Est IV.

### Main inclusion criteria

Males and females, aged 40–85, fulfilling the ACR criteria for knee OA [28], who failed to respond or were intolerant to analgesics and/or NSAIDs, having a walking pain score ranging from 3 to 8 on a 11-point Likert scale and an OARSI radiological score [29] 1 to 3, for tibio-femoral joint space narrowing (JSN).

### Main exclusion criteria

Patients under 40 or older than 85, absence of tibio-femoral JSN on standard X-rays, KOFUS (Knee OA Flare-Ups Score) > 7 [30], tibial plateau or femoral condyle bony attrition, bilateral symptomatic knee OA or any other significant musculoskeletal condition that might interfere with the assessment of the target knee pain (hip OA, active inflammatory or microcrystal rheumatic diseases, neurological diseases), excessive (≥8°) knee malalignment, HA injection(s) in the target knee within the previous 9 months, systemic or IA corticosteroids within the previous 3 months.

### Allowed concomitant treatments for OA

Paracetamol (up to 4 g/day), weak opioids, ibuprofen (up to 800 mg/day) and naproxen (up to 500 mg/day), topical NSAIDs, and symptomatic slow-acting drugs for OA if started at least 2 months before screening and not modified during the study duration. Discontinuation of analgesics was required 48 h before each evaluation visit.

### Baseline and follow-up examination

At the screening visit, age, gender, height, weight, body mass index (BMI kg.m^− 2^), medical history (previous knee HA or corticosteroid injection, disease duration), and concomitant therapies were recorded. Bilateral knee X-rays were performed including standing postero-anterior view, Lyon-schuss view [31], lateral view and skyline incidence of the patella. Investigators had to assess both OARSI score for JSN and Kellgren-Lawrence (KL) score [32] on the radiological view highlighting the most severe lesions.

At baseline and at each follow-up visit the Western Ontario and McMaster Universities Osteoarthritis Index (WOMAC) [33] and patient global assessment of pain (PGAP) score were obtained. For each of the 24 questions of the WOMAC index patients had to give a mark using a 5 point Likert scale (0 = none, 1 = mild, 2 = moderate, 3 = severe, 4 = extreme) giving a total score ranging from 0 to 20 for WOMAC pain sub-score, 0 to 68 for WOMAC function and 0 to 96 for WOMAC total.

### Treatments under study

Patients were randomized to one of the following treatment arms: HAnox-M or Bio-HA in a 1:1 ratio by blocks of 4 treatments, balanced 2:2. Both viscosupplements were supplied in 2 ml syringes containing 2 ml of HA solution and were administered by an experienced physician (orthopedic surgeon or rheumatologist), 1 week apart, for 3 consecutive weeks, into the target knee, using a 18- to 21-gauge needle, after careful removal of SF effusion if present. Injector was different from the clinical evaluator to ensure the double blind. Both patient and evaluator were blinded to the treatment allocation throughout the follow-up.

### Statistical analysis

In the present analysis patients were pooled regardless the treatment allocation, since there was no significant demographic, clinical and radiological between-group difference at baseline and month 6 [24]. Patients were classified according to body mass index (non-obese if BMI < 30 kg.m^− 2^or obese if BMI ≥ 30 kg.m^− 2^), OARSI radiological grade (1–2 versus 3) and OMERACT-OARSI response criteria (Yes/No). WOMAC pain and function scores at baseline and end-point were obtained and their decrease over the 6-month follow-up was calculated.

WOMAC pain score at baseline and end-point and WOMAC pain decrease were compared in i) non-obese versus obese patients, ii) OARSI 1–2 versus OARSI 3 JSN, iii) Non-obese/OARSI 1–2 versus obese/OARSI grade 3. The statistical analysis was performed in the total population and in the subgroup of responders.

Baseline and 6-month follow-up data are given as number, percentage or mean [95% CI]. Between-group comparisons were achieved using Mann-Whitney U test.

The statistical analysis was performed from the intent-to-treat (ITT) population. *P* values < 0.05 were considered statistically significant. XLSTAT© 2015 software (Addinsoft©, Paris, France) was used for the statistical analysis.

## Results

One hundred and sixty-six patients were analyzed: 101 (60.8%) women, mean age 65.2 [95% CI 63.7–66.8] years, average disease duration 48.7 [95% CI 38.4–59.0] months and mean BMI 27.7 [95% CI 26.9–28.5] kg.m^− 2^. Forty-seven patients were classified as obese (28.3%). Ninety-three patients (56.0%) had OARSI score 1–2 and 73 (44.0%) had OARSI score 3 (Table 1).

At baseline, the average WOMAC pain (0–20) and function scores (0–68) were 9.8 [95% CI 9.3–10.3] and 27.5 [95% CI 25.7–29.4] respectively. At baseline there was no statistical difference in WOMAC pain score according to the weight status, the radiological grade and their combination. All data and *p*-values are given in Table 2.

**Table 1:** Characteristics of patients in the intent-to-treat population

		ITT population (*N* = 205)	HANOX-M (*N* = 103)	BioHA (*N* = 102)	*p*-value
Age (years)	Mean (SD)	65.3 (10.5)	65.2 (10.1)	65.3 (10.9)	0.95
OARSI JSN	Grade 1–2 N (%)	119 (58%)	57 (55.4%)	62 (60.8%)	0.68
	Grade 3 N (%)	86 (42%)	46 (44.7%)	40 (39.2%)	
BMI (kg/m^2^)	Mean (SD)	27.59 (4.92)	27.72 (5.00)	27.47 (4.86)	0.72
WOMAC A baseline	Mean (SD)	9.7 (3.4)	9.7 (3.2)	9.7 (3.6)	0.98
WOMAC A endpoint	Mean (SD)	5.4 (4.2)	5.4 (4.0)	5.3 (4.4)	0.95

*WOMAC* Western Ontario & McMaster Universities Osteoarthritis Index: pain (range 0–20), *OARSI* OsteoArthritis Research Society International score for joint space narrowing (JSN range 0–3), *BMI* Body Mass Index (kg/m^2^)

Inversely, WOMAC function score was statistically higher in obese versus non-obese subjects and moreover in obese/OARSI 3 than in non-obese/OARSI 1–2 patients. All data and p-values are given in Table 3.

At month 6, 113 patients (68.1%) fulfilled the OMERACT-OARSI responder criteria. WOMAC pain and function scores at baseline and month 6 are given in Tables 2 and 3. WOMAC pain at month 6 was significantly lower in non-obese than in obese patients (*p* = 0,006) and in patients with OARSI grade 1–2 versus 3 (*p* = 0.02). Similarly, the decrease of WOMAC pain over time was greater in patients with OARSI grade 1–2 than in those with grade 3 (*p* = 0.008). The decrease of WOMAC pain was also greater in non-obese than in obese patients (*p* = 0.049). Unsurprisingly WOMAC pain decrease was twice greater in non-obese patients with OARSI 1–2 than in obese patients with OARSI grade 3 (*p* = 0.007). At month 6, the WOMAC pain score, was 85,7% higher in obese/OARSI 3 patients than in non-obese/OARSI1–2 subjects (*p* = 0.0001).

**Table 2:**
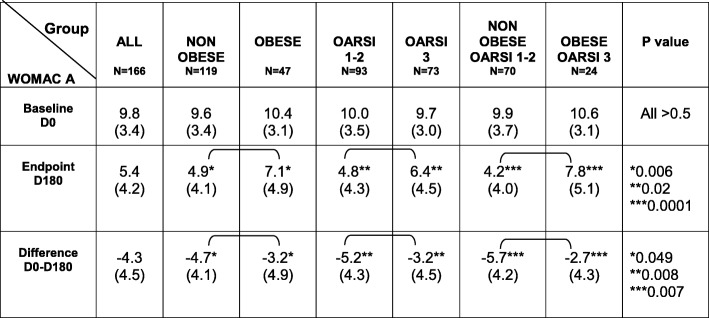
WOMAC pain score at baseline (D0) and endpoint (D180) and their difference D0-D180, according to weight status and OARSI radiological grade

*WOMAC* Western Ontario & McMaster Universities Osteoarthritis Index: pain (range 0–20), *OARSI* OsteoArthritis Research Society International score for joint space narrowing (range 0–3)

**Table 3:**
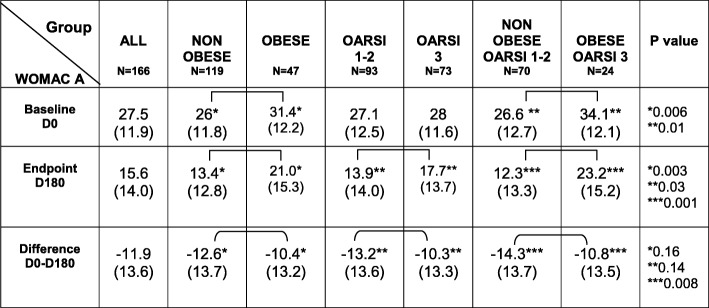
WOMAC function score at baseline (D0) and endpoint (D180) and their difference D0-D180, according to weight status and OARSI radiological grade

*WOMAC* Western Ontario & McMaster Universities Osteoarthritis Index: function (range 0–68), *OARSI* OsteoArthritis Research Society International score for joint space narrowing (range 0–3)

At month 6, as for pain, WOMAC function score was significantly higher in obese versus non-obese patients, in OARSI 3 versus OARSI 1–2 and, even more in obese/OARSI 3 than in non-obese/OARSI 1–2. A similar trend was found for WOMAC function variation (Table 3).

In the responders population (*N* = 113) there was no significant difference between subgroups (non-obese versus obese patients, OARSI 1–2 versus OARSI 3, Non-obese OARSI grade 1–2 versus obese OARSI grade 3) both for WOMAC pain score and WOMAC pain decrease (Table 4). These data suggest that, in responder patients, the magnitude of response to VS is not reduced by obesity or by severe JSN, despite the chances to be a responder are dramatically decreased in case of obesity and/or advanced radiological score.

**Table 4:** WOMAC pain score at baseline (D0) and endpoint (D180) according to weight status and OARSI radiological grade, in patients fulfilling the OMERACT-OARSI response criteria

Group WOMAC A	ALL *N* = 113	NON OBESE *N* = 91	OBESE *N* = 22	OARSI 1–2 *N* = 73	OARSI 3 *N* = 40	NON OBESE OARSI 1–2 *N* = 61	OBESE OARSI 3 *N* = 10	*P* value
Baseline D0	9.9 (3.3)	9.8 (3.4)	10.1 (2.6)	9.9 (3.5)	9.8 (2.9)	9.8 (3.7)	10.0 (3.1)	All > 0.5
Endpoint D180	3.3 (2.7)	3.3 (2.8)	3.2 (2.2)	3.2 (2.7)	3.4 (2.6)	3.2 (2.8)	3.4 (2.7)	All > 0.5
Difference D0-D180	−6.5 (3.2)	−6.9 (2.8)	−6.7 (3.4)	− 6.4 (2.6)	− 6.4 (2.6)	−6.6 (3.4)	−6.7 (2.3)	All > 0.5

*WOMAC* Western Ontario & McMaster Universities Osteoarthritis Index: pain (range 0-20), *OARSI* OsteoArthritis Research Society International score for joint space narrowing (range 0-3)

## Discussion

In a previous work [24] we showed that the rate of success of knee VS, assessed by the percentage of patients who fulfilled the OMERACT-OARSI response criteria at month 6, was significantly lower in patients with BMI ≥ 30 than in those with normal weight or moderate overweight, in those with advanced radiological stage of OA, and moreover in subjects who combined the two risk factors.

The present results confirm that both the decrease of symptoms over time and the level of pain 6 months after HA injections are significantly different according to the radiographic severity and the patient’s weight status. Nevertheless, in our study, the magnitude of response to VS was not significantly altered in obese subjects with advanced JSN, who fulfilled the OMERACT-OARSI response criteria. This suggests that a particular sub-group of patients with both obesity and severe disease may greatly benefit from VS.

The importance of the radiological severity on VS efficacy is well documented and has been many times underlined [21–24]. From the data of the FLEXX and FLEXX extension trials, Altman et al. [23] showed that the decrease of WOMAC pain score was significantly greater in subjects with KL grade II than in those with KL grade III. However the severity of JSN should not be an absolute contraindication to VS. A workgroup of clinical experts who developed an Appropriate Use Criteria for viscosupplementation in knee OA [34] concluded that, despite the insufficient evidence to advise VS in patients with severe OA, the collective experience of the task force suggested not to discredit VS in such patients who may benefit from the treatment, particularly when other pharmacological or surgical modalities are contra-indicated. In the consensus statement on VS with HA for the management of OA, Henrotin et al. [20] agreed with the issue that VS may also be helpful in advanced stages of knee OA, considering that, in patients with KL IV, VS could be proposed as an adjunctive therapy to relieve pain, particularly in patients who do not want or cannot, because of co-morbidities, undergo surgery. It also should be stressed that HA has a NSAIDs sparing effect, which can be useful in frail and old patients with severe disease [35]. Our results are in line with the opinion of these authors. They showed that 6 patients out of 10 with grade 3 JSN greatly benefited from the treatment (mean decrease of pain − 6.4 ± 2.5), similarly to patients with less severe OA (− 6.7 ± 2.5).

Our previous study also showed a strong relationship between obesity and risk of VS failure [24]. Similar findings were reported in an open-label trial [36], in patients with knee OA treated with a single IA injection of a mannitol-modified cross-linked HA, that showed that the percentage of subjects reaching the Patient Acceptable Symptom state threshold [37] was significantly lower in obese than in non-obese subjects. The present analysis confirms that the magnitude of the pain improvement was much greater in patients with normal weight or moderate overweight than in obese subjects. It is however important to underline that, in our study, obese patients who fulfilled the OMERACT-OARSI response criteria experienced a relief of pain identical to that of non-obese subjects (− 6.9 ± 2.8 versus − 6.5 ± 3.2; *p* > 0.5). It is therefore possible to obtain a significant pain improvement in obese patients, even if the chances of achieving an excellent result are lower than in normal weight subjects.

We previously showed a very low rate of response (about 40%) in patients who cumulated obesity and severe JSN. Interestingly similar trends have been reported after IA corticosteroid injection, in a cohort of 100 patients with knee OA [38].

In our study the decrease of pain was two-fold greater in non-obese patients with mild to moderate JSN than in obese patients with OARSI grade 3 (− 58.2% versus − 28.6%).

However, in the sub-group of obese patients with severe JSN who were classified as “responders” according to the OMERACT-OARSI criteria, the WOMAC pain score at month 6 and the decrease of pain over time (3.3 ± .9 and − 6.6 ± 3.4 respectively) did not differ from that of patients with no risk factor (3.2 ± 2.8 and − 6.7 ± 2.3 respectively). These results raise a crucial issue regarding the reasons for the efficacy or non-efficacy of HA injections in patients with severe OA and/or obesity. It is currently unknown whether patients with different pain phenotypes may respond differently to viscosupplementation. For example, in some patients synovitis may be the main cause of pain, while in others the bone origin may be predominant [39, 40]. Moreover, pain in OA is usually viewed as of nociceptive origin, but it is now demonstrated that a significant percentage of knee OA patients experience neuropathic pain [40]. Based on the known mechanisms of action of HA, one can hypothesize that individuals with predominantly nociceptive pain might benefit more from VS than those with neuropathic pain. Further studies, investigating the role of phenotypic characteristics of pain on the results of VS, with a particular focus on the role of obesity [41], are needed to answer this question.

Another interesting data to point out, is that WOMAC function was significantly higher in obese than in non-obese subjects, regardless of the level of pain or of the X ray grade. It is obvious that obese patients may give higher scores to questions regarding the difficulties to get in or out of the bathtub or the bed, and to put or remove socks or to do housework. This must be known and taken into account by physicians when assessing the functional status of patients with knee OA.

Our study has limitations, especially because it is a post-hoc analysis of a trial, which has not been designed for this purpose. Thus, only 166 of the 205 patients from the intent-to-treat population had full data allowing to include them in the post-hoc analysis. Furthermore the sample of patients with both obesity and severe JSN was small, limiting the power of the analysis. To explain the lack of effectiveness of VS in obese patients, we cannot exclude a bias due to the increased risk of needle misplacement, leading to extra-articular HA injection related to larger subcutaneous adipose tissue, in obese subjects [42]. This concern is frequent in daily clinical practice since knee HA injections are usually achieved without the help of an imaging guidance. Further studies, designed to compare the results of VS performed with or without imaging guidance, are needed.

## Conclusion

Our results do not confirm our previous conclusions that recommended not performing viscosupplementation in obese patients with anatomically severe knee OA. Although the chances of being a responder are much reduced in these patients, the benefit of patients who respond to treatment is similar to that of subjects who do not have these two risk factors of treatment failure. This knowledge should avoid of not recommending a therapeutic option that benefit a substantial number of patients, in a chronic and debilitating pathology in which few effective and well-tolerated treatments are available.

## Data Availability

Data from the HAV-2012-1 trial are accessible at Laboratoire de Rhumatologie Appliquée, 19 place Tolozan, F-69001Lyon, France.

## Abbreviations

BMI: Body Mass Index
HA: Hyaluronic Acid
IA: Intra Articular
ITT: Intent-to-Treat
JSN: Joint Space Narrowing
KL: Kellgren-Lawrence
KOFUS: Knee Osteoarthritis Flare-Up Score
NSAID: Non Steroidal Anti Inflammatory Drug
OA: OsteoArthritis
OARSI: OsteoArthritis Reseach Society International
OMERACT: Outcome Measures in Rheumatology
SF: Synovial Fluid
VS: ViscoSupplementation
WOMAC: Western Ontario MAC Master University
